# Insights into Hepatocellular Carcinoma: From Pathophysiology to Novel Therapies

**DOI:** 10.3390/ijms25084188

**Published:** 2024-04-10

**Authors:** Daniela Gabbia, Sara De Martin

**Affiliations:** Department of Pharmaceutical and Pharmacological Sciences, University of Padova, 35131 Padova, Italy; sara.demartin@unipd.it

Hepatocellular carcinoma (HCC), the most common primary liver cancer, accounts for 830,180 related deaths worldwide in 2020, according to GLOBOCAN, representing the fourth leading cause of cancer-related death, with a five-year survival rate of about 18% for advanced stage, and the second leading cause in men of cancer-related mortality worldwide [1]. Liver cancer is characterized by a high fatality rate; indeed, incidence and mortality age-standardized rates are quite comparable, so its treatment is considered a global clinical challenge [2].

This Special Issue would like to offer an update on novel studies able to elucidate the pathological mechanisms of the neoplastic transition, which can be related to the different HCC etiologies. Collectively, the final aim of these studies is the identification of novel pharmacological targets, therapeutic options, and/or biomarkers that could potentially be exploited in the field of hepatic cancer.

HCC development usually occurs in the context of chronic liver damage culminating in cirrhosis. The triggers of the damage are chronic viral hepatitis (HBV and HCV), alcohol abuse, and metabolic dysfunction-associated steatotic liver disease (MASLD) or steatohepatitis (MASH). Approximately 80% of HCC cases originate from cirrhotic livers, and one-third of cirrhotic patients, irrespective of the etiology, are expected to develop HCC. Taken together, these peculiar features, aside from highlighting the intricate interplay between chronic liver diseases and the onset of HCC, emphasize the critical need for early detection and intervention strategies. Many hepatic cell types are involved in controlling fibrosis progression. Emerging evidence suggests that liver sinusoidal endothelial cells (LSECs) regulate the molecular composition of circulating blood, thereby controlling the homeostasis and functions of other organs, through a variety of scavenger receptors, e.g., Stabilin-1 (Stab1) and Stabilin-2 (Stab2). A study by Krzistetzko et al. observed that Stabilin deficiency may increase collagen depositions under homeostatic conditions. Moreover, after an experimental dietary challenge, the hepatic content of the Stabilin ligand TGFBi is differently altered in Stabilin-deficient mice, suggesting that the scavenger functions of LSECs are differentially affected in these mice. These findings suggest the evaluation of anti-Stabilin therapies, which are currently undergoing clinical evaluation for other diseases, for the improvement of hepatic fibrosis [3]. 

The pathogenesis of HCC is a multifaceted and complex process often triggered by liver injury and the consequent inflammation. Chronic liver inflammation ignites the fibrotic response, which gradually advances to cirrhosis, a condition where liver tissue architecture is disrupted. Persistent inflammation and cirrhosis set the stage for precancerous conditions, fostering the accumulation of genetic and epigenetic alterations over time, ultimately culminating in HCC development. In this context, the role of the tumor microenvironment (TME) is pivotal for disease progression. In their review, Kotsari and collaborators presented an overview of the role of the HCC immune microenvironment, with a particular focus on the cellular constituents, the current therapies targeting TME, and the potential of immunotherapy in the clinical setting [4]. The modulation of the TME is also the topic of the work of Radić and colleagues, who described the role of LOXL2, a copper-dependent amine oxidase that may foster the formation of premetastatic niches and metastasis and help the epithelial–mesenchymal transition, angiogenesis, and vasculogenic mimicry [5].

For over a decade, sorafenib represented the primary therapeutic option for advanced HCC. However, in the last few years, regulatory agencies have approved several immunotherapy regimens as first- and/or second-line options, able to improve patients’ survival. On the other side, many efforts have been devoted to the identification of novel signaling pathways that may be “druggable” and exploited to cure or prevent HCC, e.g., those involved in the modulation of the antitumoral immune response. Despite these efforts, HCC remains a challenging global medical need. Moreover, the criteria for predicting prognosis and deciding the treatment modalities of the most widely used classification, i.e., the Barcelona Clinic Liver Cancer (BCLC), are not always in line with patient-tailored therapeutic decision-making. For this reason, many studies suggest the introduction of a novel approach based on “converse therapeutic hierarchy” [6], in which the advanced treatment modalities may be better integrated to achieve patient-tailored management. In this context, we included a retrospective study enrolling 115 HCC patients at advanced stage who received lenvatinib (LEN) combined with transcatheter intra-arterial therapy (TIT) after propensity score matching observed that this combination therapy may improve prognosis with respect to lenvatinib monotherapy in advanced HCC patients, particularly those <75 years of age and with modified albumin bilirubin (m-ALBI) grade 1 [7].

The pilot study by Fasolato and collaborators investigated the predictive value of cytokine profiles in HCC patients, evaluating 45 proteins in plasma and ascitic fluids collected from 44 cirrhotic patients with or without HCC of different etiologies [8]. They observed that low levels of IL-5 and granulocyte–macrophage colony-stimulating factor in ascitic fluids, high plasma levels of eotaxin-1 and hepatocyte growth factor and stromal-cell-derived factor 1α, as well as HCC, HBV, or HCV, were associated with a poor prognosis and decreased survival, thus may be predictive of the patient’s prognosis.

HCC incidence is also increased in thalassemic patients, where hepatic iron overload may lead to liver fibrosis, cirrhosis, and finally tumor development. The review by Lin et al. discusses the pathogenesis and the current perspective on HCC surveillance and management in patients with thalassemia [9]. Another disease that may increase the risk for HCC development is primary biliary cholangitis (PBC), a chronic autoimmune liver disease characterized by the immune-mediated destruction of bile ducts. Floreani and collaborators summarize the state of the art regarding the molecular alterations shared by PBC and HCC and the risk factors that may be predictive for HCC in PBC patients [10].

Due to its high morbidity, mortality, and growing incidence, HCC is a hot topic in the oncology scenario. The identification of feasible biomarkers and specific targets that may be used for achieving an early diagnosis of new cases is crucial, along with the improvement of the available therapeutic options, for example, by setting up new combination therapies, to ameliorate HCC prognosis and patient survival.
